# Inhibition of DNA Glycosylases via Small Molecule Purine Analogs

**DOI:** 10.1371/journal.pone.0081667

**Published:** 2013-12-09

**Authors:** Aaron C. Jacobs, Marcus J. Calkins, Ajit Jadhav, Dorjbal Dorjsuren, David Maloney, Anton Simeonov, Pawel Jaruga, Miral Dizdaroglu, Amanda K. McCullough, R. Stephen Lloyd

**Affiliations:** 1 Center for Research on Occupational and Environmental Toxicology, Oregon Health and Science University, Portland, Oregon, United States of America; 2 National Center for Advancing Translational Sciences, National Institutes of Health, Rockville, Maryland, United States of America; 3 Biomolecular Measurement Division, National Institute of Standards and Technology, Gaithersburg, Maryland, United States of America; 4 Department of Molecular and Medical Genetics, Oregon Health and Science University, Portland, Oregon, United States of America; University of Massachusetts Medical School, United States of America

## Abstract

Following the formation of oxidatively-induced DNA damage, several DNA glycosylases are required to initiate repair of the base lesions that are formed. Recently, NEIL1 and other DNA glycosylases, including OGG1 and NTH1 were identified as potential targets in combination chemotherapeutic strategies. The potential therapeutic benefit for the inhibition of DNA glycosylases was validated by demonstrating synthetic lethality with drugs that are commonly used to limit DNA replication through dNTP pool depletion via inhibition of thymidylate synthetase and dihydrofolate reductase. Additionally, NEIL1-associated synthetic lethality has been achieved in combination with Fanconi anemia, group G. As a prelude to the development of strategies to exploit the potential benefits of DNA glycosylase inhibition, it was necessary to develop a reliable high-throughput screening protocol for this class of enzymes. Using NEIL1 as the proof-of-principle glycosylase, a fluorescence-based assay was developed that utilizes incision of site-specifically modified oligodeoxynucleotides to detect enzymatic activity. This assay was miniaturized to a 1536-well format and used to screen small molecule libraries for inhibitors of the combined glycosylase/AP lyase activities. Among the top hits of these screens were several purine analogs, whose postulated presence in the active site of NEIL1 was consistent with the paradigm of NEIL1 recognition and excision of damaged purines. Although a subset of these small molecules could inhibit other DNA glycosylases that excise oxidatively-induced DNA adducts, they could not inhibit a pyrimidine dimer-specific glycosylase.

## Introduction

The DNA base excision repair (BER) pathway has evolved to respond to ongoing challenges to genome stability that are posed by oxidation, alkylation, and deamination of DNA bases. In humans, the initiation of BER of DNA damage arising from oxidative stress occurs through the collective activities of the DNA glycosylases NEIL1, NEIL2, NEIL3, OGG1, and NTH1 (reviewed in [1]). Through a series of sequential biochemical steps, these enzymes flip the damaged nucleotide to an extrahelical position and catalyze removal of the damaged base through glycosyl bond scission, followed by phosphodiester bond breakage. Of the various oxidatively induced DNA lesions, NEIL1 has distinct substrate preference for ring-fragmented purine derivatives such as 4,6-diamino-5-formamidopyrimidine (FapyAde) and 2,6-diamino-4-hydroxy-5-formamidopyrimidine (FapyGua), and for a subset of ring-saturated pyrimidines, including thymine glycol (Tg) [2], [3], [4], [5], [6]. It also removes oxidation products of 7,8-dihydro-8-oxo-guanine (8-oxo-Gua), such as spirodihydantoin (Sp) and guanidinohydantoin (Gh) from oligodeoxynucleotides [3]. OGG1 primarily recognizes 8-oxo-Gua and FapyGua [7], while together, NTH1 and NEIL2 remove the majority of ring-saturated pyrimidines [8], [9]. Similar to NEIL1, NEIL3 is also specific for FapyAde and FapyGua, along with 8-hydroxyadenine and some pyrimidine-derived lesions such as Tg, 5-hydroxycytosine and 5-hydroxy-5-methylhydantoin [10].

Although BER is critical for genome stability, there are circumstances in which the inhibition of this repair pathway as part of a synthetic lethality strategy has proven to be efficacious in the treatment of certain cancers. This therapeutic approach has been used effectively in treating BRCA1/2- or PTEN-deficient tumors (defective in homologous recombination) with inhibitors of PARP1, another key enzyme in the BER pathway [11], [12], [13], [14]. In order to further identify and exploit other points in the BER pathway, Taricani et al [15] conducted an investigation to identify gene-specific pathways that would function as synthetic lethal partners with DNA glycosylases as the target for combination chemotherapy, and chemotherapeutic agents that function through depletion of cellular dNTP pools. Specifically, a key enzyme in thymidine biosynthesis is thymidylate synthetase (TS), which is responsible for the reductive methylation of dUMP by N5, N10-methlyene tetrahydrofolate to form dTMP and dihydrofolate [16], [17]. Drug inhibitors that target the TS pathway are widely used in the treatment of a variety of human cancers including ovarian, gastric, colorectal, pancreatic, breast, and head and neck. These are generally folate-based analogs, but nucleotide-based inhibitors are also used [18], [19], [20], [21]. Due to their targets of action, these inhibitors are primarily toxic in the S-phase of the cell cycle through the depletion of intracellular dTTP, stalling of DNA replication and increasing dUMP incorporation into DNA. Commonly used inhibitors of TS are raltitrexed (Tomudex ®; RTX) and nolatrexed (NOL), while inhibitors of dihydrofolate reductase (DHFR) that result in depletion of tetrahydrofolate, with associated decreases in purine and pyrimidine synthesis, include methotrexate (MTX) and aminopterin (AMT). Taricani et al showed that although siRNA-mediated reduction in several DNA glycosylases in an osteosarcoma cell line, including NEIL1 and OGG1 (and to a lesser degree NTH1, MPG, SMUG1, and TDG) had no effect on cytotoxicity, when used in combination with minimally toxic doses of MTX, AMT, RTX and NOL, these combinations resulted in synergistic increases in γH2AX positive cells [15]. For NEIL1-depleted cells, treatments of MTX, AMT, RTX and NOL resulted in increased cytotoxicity of 10-fold, 7-fold, 9-fold and 5-fold, respectively. In addition, loss of NEIL1 function has also been shown to be synthetically lethal with the disruption of the Fanconi anemia DNA repair pathway, in which the disease is characterized by a deficiency in repair and tolerance of interstrand DNA cross-links [22]. To discover genes that may exhibit synthetic lethality with FancG, cells that were deficient in this gene were screened by various siRNA treatments [22]. In addition to the discovery of synthetic lethality with ATM, knockdown of *Neil1* message also conferred lethality to FancG cells.

Based on these discoveries, we have developed a research strategy to discover inhibitors of DNA glycosylases, with an ultimate goal being to develop and utilize high affinity, high specificity small molecules in combination protocols to synergistically enhance the therapeutic efficacy of other cancer treatments, including, but not limited to TS and DHFR inhibitors. As a proof-of-principle, we have chosen to implement the first phase of these investigations by screening for small molecule inhibitors of NEIL1. Strategies that create synthetic lethal relationships between disparate pathways have the potential to be far less toxic and debilitating to cancer patients, while still achieving a high therapeutic outcome. Together, these possible applications indicate the value of clinical exploitation of inhibitors of DNA glycosylases.

## Materials and Methods

### Reagents

Tris-HCl, Tween-20, EDTA, NaCl, KCl, MgCl_2_, and DTT were purchased from Sigma-Aldrich (St. Louis, MO). Dimethyl sulfoxide (DMSO), urea, acrylamide, bisacrylamide and bovine serum albumin (BSA) were purchased from Fisher Scientific (Pittsburgh, PA). Black 384-well and 1,536-well plates were purchased from Corning (Corning, NY). DNA ladder was purchased from New England BioLabs (Ipswich, MA). 4,6-Diamino-5-formamidopyrimidine-^13^C, ^15^N_2_ (FapyAde-^13^C, ^15^N_2_) and 2,6-diamino-4-hydroxy-5-formamidopyrimidine-^13^C, ^15^N_2_ (FapyGua-^13^C, ^15^N_2_) were purchased from Cambridge Isotope Laboratories (Andover, MA).

### Small molecule inhibitors

The screening collection consisted of the LOPAC^1280^ collection from Sigma–Aldrich (1280 compounds) and cherry-picked purine analogs from the ∼400,000-member Molecular Libraries Small Molecule Repository (MLSMR) collection. Additional hit compounds were purchased from Sigma-Aldrich.

### Glycosylase enzymes

FPG and UDG were purchased from New England BioLabs. Human NEIL1 was expressed and purified as previously reported [4]. Human OGG1 and NTH1 expression constructs were generous gifts of Dr. Gregory Verdine (Harvard University, Cambridge, MA) and Dr. Susan Wallace (University of Vermont, Burlington, VT), respectively. His-tagged OGG1 and NTH1 proteins were expressed and purified using the same methods as described for NEIL1 [4]. T4 pyrimidine dimer DNA glycosylase (T4-pdg) was purified as previously described [23].

### Oligodeoxynucleotide substrates

**Figure 1:**
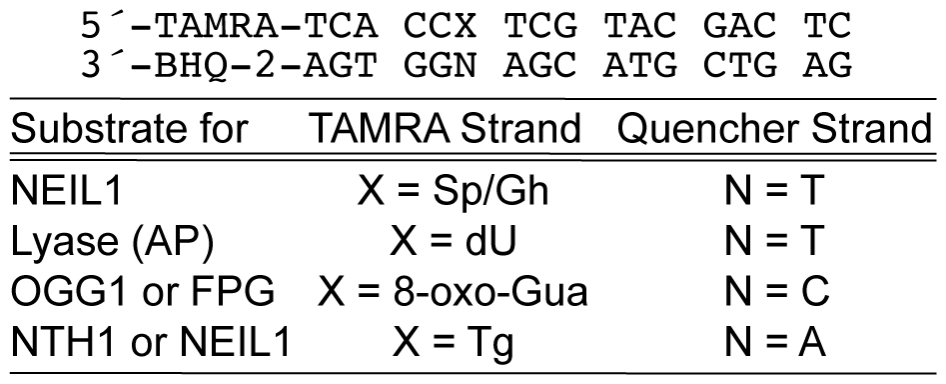
Design of oligonucleotide substrates.

Oligodeoxynucleotides were purchased from Biosearch Technologies, Inc. (Novato, CA), Integrated DNA Technologies (Coralville, IA) or generously provided by Dr. Carmelo J. Rizzo (Department of Chemistry, Vanderbilt University, Nashville, TN). The double-stranded DNA substrate used in the fluorogenic assay was prepared from two oligodeoxynucleotides: the quencher (Black Hole Quencher 2 [BHQ-2]) strand, and fluorescent reporter (TAMRA) strand (Figure 1). 8-oxo-Gua, Tg, and 2′-deoxyuridine (dU) were incorporated synthetically, while Sp and Gh, were obtained through a secondary oxidation as previously reported [24]. Briefly, a 500 µL solution of the oligodeoxynucleotide containing 8-oxo-Gua (12 µM) in PBS (pH 7.2) was treated with NaIrCl_2_ (100 µM) at 65°C for 30 min, then quenched with EDTA (160 µM) and purified by centrifugal filtration. DNA containing a site-specific abasic (AP) lesion was created by treating fully duplex DNA containing a site-specific uracil with UDG. Annealing of the oligodeoxynucleotide components was performed in 20 mM Tris-HCl, pH 8.0, 100 mM KCl by first incubating 1.5 molar equivalents of the quencher strand with one molar equivalent of the reporter fluorescent strand at 95°C for 5 min, followed by gradual cooling to room temperature. The choice of placing a T opposite the Sp/Gh site was made based on the most efficient kinetic data as reported by the David laboratory [25]. The double-stranded substrate was stored at 4°C until use.

### Combined glycosylase/AP lyase cleavage assays

The 384-well fluorogenic assay was carried out in a 20 µL reaction mixture in 20 mM Tris-HCl pH 8.0, 100 mM KCl, and 0.1% BSA, with 5 nM active enzyme and 50 nM substrate. Possible inhibitors were incubated with the enzyme for at least 5 min at room temperature in a black, low-volume 384-well plate before addition of an equal volume of substrate to start the reaction. The plates were spun for 30 s at 1000 rcf to remove bubbles. The reactions were incubated at 37°C and fluorescence data collected on an Infinite M200 (Tecan, Mannedorf, Switzerland) equipped with standard optics (excitation filter 525 nm, and emission filter 598 nm). For the gel-based assay, reactions were initiated as described for the fluorogenic assay, but quenched with 20 µL 95% formamide at 20 min. The products were separated by electrophoresis through a 15% polyacrylamide gel containing 8 M urea and visualized by a Typhoon scanner (GE Life Sciences, Piscataway, NJ). The fraction of cleaved product was measured using ImageQuant software and analyzed using GraphPad Prism 6.

T4-pdg activity was assayed by a plasmid nicking assay with minor modifications of a previously described method [26], [27]. Supercoiled pBR322 plasmid was irradiated with 254 nm ultraviolet (UV) light (100 µW/cm^2^) for 98 sec to produce approximately 10 thymine dimers per plasmid molecule [28]. Irradiated plasmid (10 nM) was then incubated in the presence or absence of inhibitors with 130 pM active T4-pdg, as assessed by NaCNBH_4_ trapping [27]. Reactions were started by addition of DNA, incubated for 15 min at 37°C in reaction buffer (10 mM Tris pH 8.0, 1 mM EDTA, 125 mM NaCl, 100 µg/ml BSA), and stopped by the addition of SDS to a final concentration of 1%. Plasmid DNAs (form I, II and III corresponding to supercoiled, nicked circular and linear, respectively) were resolved on a 0.9% agarose gel and visualized with 0.5 µg/ml ethidium bromide. Gel images were captured on a ChemiImager 5500 (Alpha Innotech, Santa Clara, CA) gel documentation system and densitometry was performed using Image J software. Percent remaining form I DNA was calculated by applying a factor of 1.42 to the form I band intensity and then dividing form I by the sum of form I, II and III. The number of nicks per molecule were estimated based on a Poisson distribution, in which nicks per molecule  =  -ln (mass fraction of form I DNA remaining), and inhibition of activity was measured as the percentage reduction in the number of nicks per plasmid molecule compared to reactions without inhibitor.

### High-throughput screen in 1536-well format

The fluorogenic assay was miniaturized to 4 µL in 1536-well black solid plates by optimization of the enzyme and Sp/Gh substrate concentrations. Briefly, 3 µL of 25 nM NEIL1 were dispensed into each well of a 1536-well plate (except buffer in columns 3 and 4 as a negative control). Compounds (DMSO solutions) and control (DMSO only) (23 nL) were transferred to the plate via Kalypsys pintool. The plate was incubated for 15 min at room temperature, and then 1 µL of the Sp/Gh-containing DNA substrate (75 nM final concentration) was added to start the reaction. The plate was immediately transferred into the ViewLux reader for the kinetic fluorescence read. Initial rate data were normalized against uninhibited and no-enzyme controls and the resulting percent activities as a function of compound concentration were fitted using the Hill equation.

### Cell survival assay

Wild-type or *Neil1*-KO MEFs were plated in a 96-well opaque walled dish (2000 cells/well) and incubated for 18 h to allow for attachment. Inhibitor (80 µL) diluted in DMEM was added to the cells at various concentrations ranging from 0–20 µM, and incubated for an additional 72 h. Cell viability was measured using the CellTiter Glo reagent (Promega, Madison, WI) and was prepared as per the manufacturer's instructions, with 50 µL of the reconstituted reagent added to each well. Cells were incubated for 10 min on an orbital shaker to induce cell lysis. Survival was measured by luminescence on a microplate reader (TECAN) and normalized to no-treatment control.

### Measurement of the activity of NEIL1 by gas chromatography/tandem mass spectrometry

The enzymatic activity of NEIL1 was measured using gas chromatography/isotope-dilution tandem mass spectrometry (GC-MS/MS) and calf thymus DNA samples γ-irradiated at 20 Gy as described [29], [30]. Aliquots of FapyAde-^13^C,^15^N_2_ and FapyGua-^13^C,^15^N_2_ were added as internal standards to 50 µg of DNA samples. After drying in a SpeedVac, DNA samples were dissolved in 50 µL of an incubation buffer consisting of 50 mM phosphate buffer (pH 7.4), 100 mM KCl, 1 mM EDTA, and 0.1 mM dithiothreitol, and then incubated with 2 µg NEIL1 for 1 h at 37°C without any inhibitor, DMSO alone, or with 10 µL of an inhibitor solution in DMSO (2 mM). After incubation, 150 µL of cold ethanol was added. The samples were kept at −20°C for 1 h and then centrifuged with 14,000 g for 30 min at 4°C. The supernatant fractions were separated and ethanol removed in a SpeedVac under vacuum. The samples were then frozen in liquid nitrogen and lyophilized overnight. To fully remove DMSO that would interfere with GC-MS/MS analysis, 500 µL water were added to the samples followed by lyophilization overnight. This procedure was repeated twice. Dried samples were derivatized and analyzed by GC-MS/MS as described [29], [30]. For each data point, three independently prepared samples were used.

### Intercalation assay

To test whether potential inhibitors could exert their inhibitory effect via DNA intercalation, 10 µL reactions containing 500 ng of a 100-bp DNA ladder were incubated at room temperature for 20 min with 40 µM of the selected compound. Ellipticine, a compound that is known to intercalate into duplex DNA was used as a control compound at a final concentration of 40 µM. Samples were resolved on a 1% agarose gel in 0.5X Tris-borate EDTA running buffer at 120 V for 2 h. Shifts in gel mobility were visualized by post staining with ethidium bromide and images were captured on a ChemiImager 5500 (Protein Simple, Santa Clara, CA).

## Results and Discussion

### Development of a high-throughput assay for combined DNA glycosylase/AP lyase activities

The overall design of a high-throughput, fluorescence-based assay to detect small molecule inhibitors of DNA glycosylases that have an associated AP lyase activity was patterned after an assay previously developed for the detection of inhibitors of apurinic/apyrimidinic endonuclease 1 (APE1) [31] and is also similar in principle to assays designed to detect inhibitors of DNA polymerases [32], [33]. This basic strategy contrasts with the uracil-directed, tethered ligand strategy developed by the Stivers group for discovery of UDG2 inhibitors [34], or the hairpin strategy utilized by the Iwai group for probing cellular BER activity [35]. While these models were efficient in the identification of novel inhibitors [36], [37] and allowed for detection of glycosylase activity, neither approach was designed or adapted to probe large libraries of small molecules as part of a high-throughput screen.

**Figure 2:**
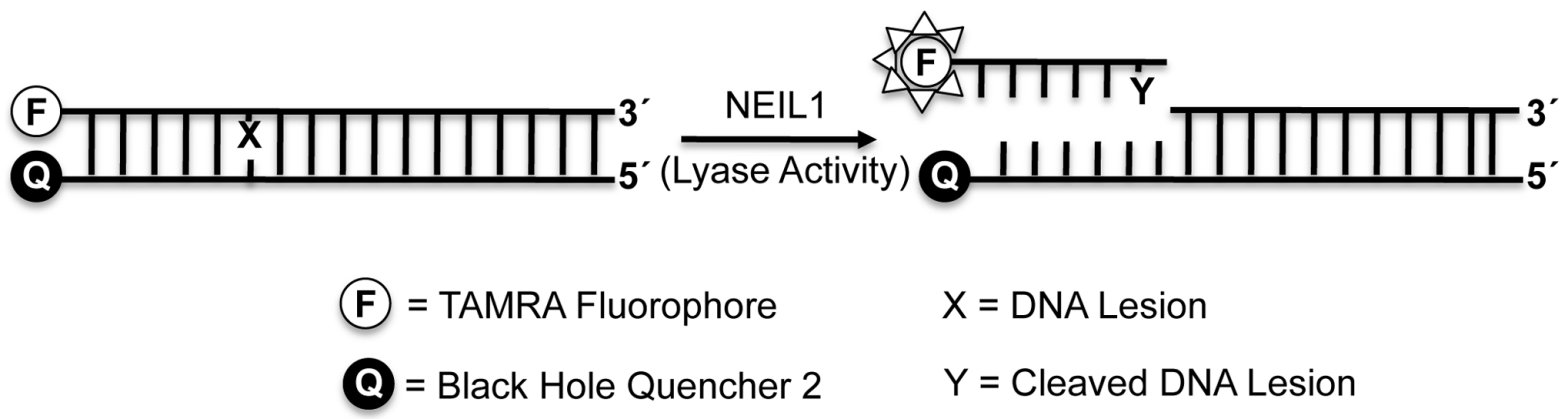
Schematic representation of fluorescence-based assay for high-throughput screen.

To create a screening assay for inhibitors that can be generalized to most DNA glycosylases, we chose to reduce this to practice using NEIL1, an enzyme which possesses both glycosylase and AP lyase activities. Although the specificities of DNA glycosylases are dictated by the range of DNA base damages that can be recognized and removed by cleavage of the respective glycosyl bond, this incision does not result in sufficient DNA structural changes that can be detected by changes in fluorescence. The assay that was developed exploits the lyase step which follows glycosidic bond cleavage in the bifunctional DNA glycosylases and results in strand cleavage that can be detected via lowering of the melting temperature of the fluorescent-containing DNA molecule (Figure 2). In principle, this assay can be modified for any glycosylase that has an associated AP lyase activity, using appropriately designed DNA substrates. Briefly, in this assay, a short synthetic oligodeoxynucleotide containing a site-specific lesion (70∶30 mixture of Sp:Gh adducts) and a 5′-TAMRA fluorophore was hybridized to a complementary DNA containing a 3′-BHQ-2 molecule. After annealing the strands, the TAMRA and the BHQ-2 were spatially adjacent. Although this duplex produced only background levels of fluorescence, following addition of a bifunctional glycosylase, cleavage at the site of the lesion reduced the melting temperature of the fluorescently-labeled fragment. The incised DNA fragment containing the TAMRA diffused into bulk solution and its fluorescent signal was detected. In contrast, addition of small molecule inhibitors of the glycosylase would prevent incision such that no fluorescent signal would be produced and the solution would remain dark (Figure 2).

**Figure 3:**
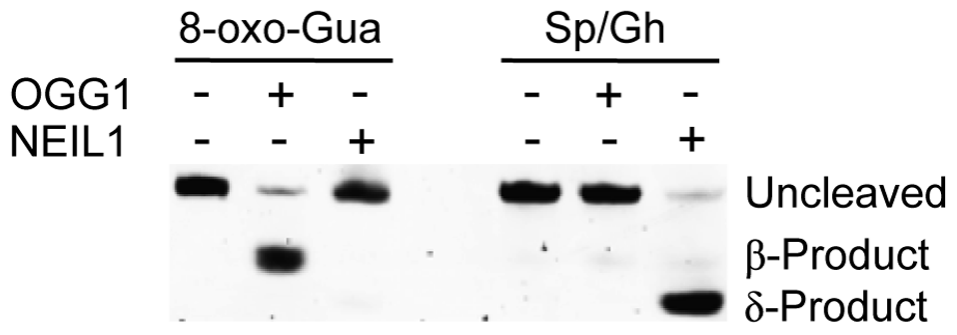
Electrophoretic mobility of products after cleavage with the glycosylase-lyases OGG1 and NEIL1. Left, before secondary oxidation, and right, after secondary oxidation.

Specifically for NEIL1, a 17-mer oligodeoxynucleotide containing an 8-oxo-Gua lesion positioned 6 nucleotides downstream of a 5′-TAMRA fluorophore was subjected to oxidation to produce DNAs containing both Sp and Gh, two modified nucleotides that have been previously established to be excellent substrates for NEIL1 [24]. HPLC analyses of the products revealed that the secondary oxidation reaction had proceeded to completion, producing a mixture (70∶30 ratio) of Sp and Gh. The lesion strand was hybridized to a complementary DNA containing a 3′-BHQ-2 [38]. Incubation of this substrate with NEIL1, but not OGG1, resulted in the incision of the substrate DNA. These data verified that the fluorescently-labeled DNA was an appropriate substrate for NEIL1 (Figure 3).

**Figure 4:**
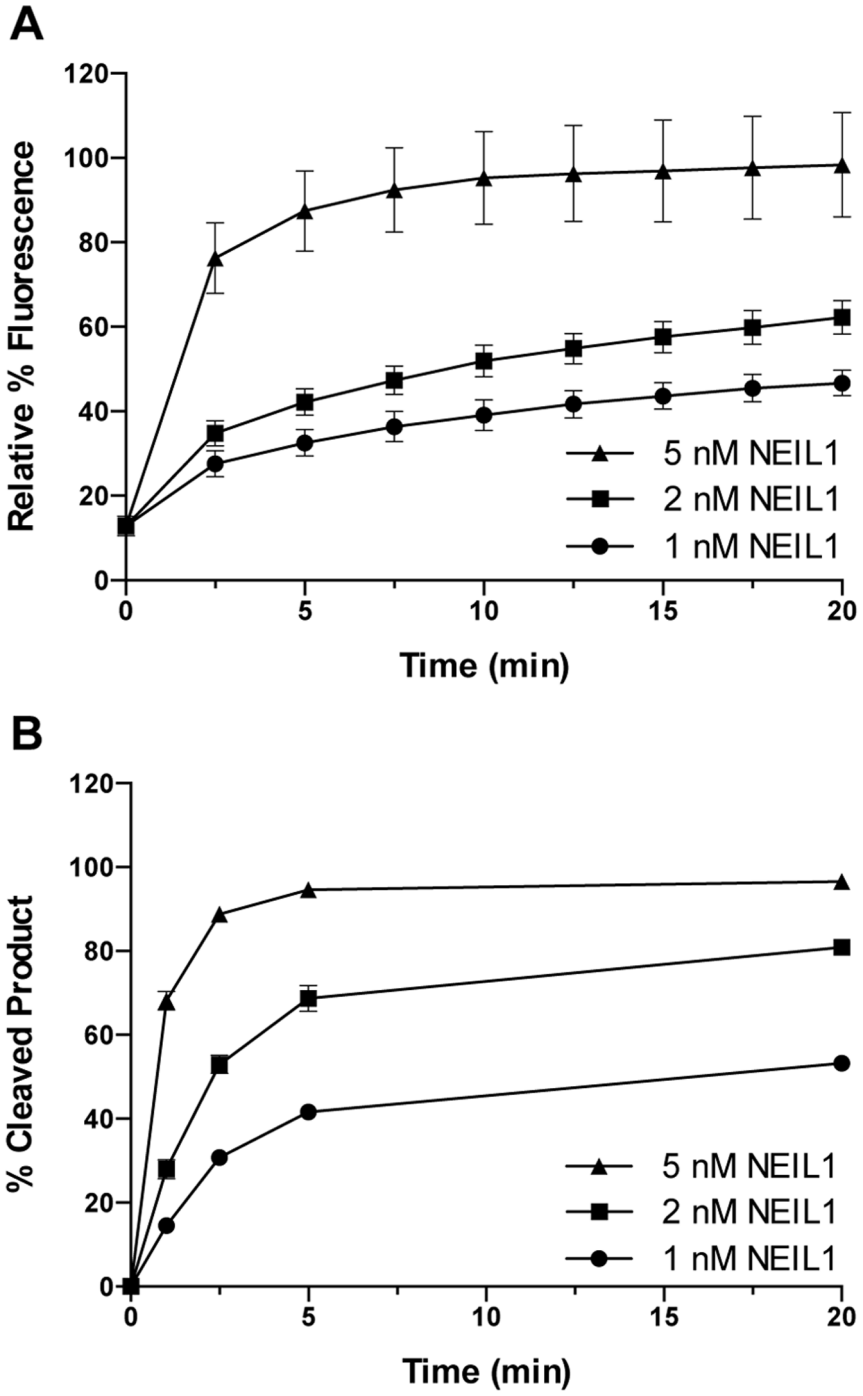
Cleavage of Sp/Gh substrate by NEIL1. A) Fluorescent signal observed when Sp/Gh substrate was incubated with various concentrations of NEIL1. B) Cleavage observed by gel electrophoresis when Sp/Gh substrate was incubated with various concentrations of NEIL1.

As an initial reduction-to-practice, experiments were optimized using a 384-well platform, with a final total reaction volume of 20 µL, in which varying concentrations of NEIL1 were added to solutions of lesion-containing substrate DNA. After mixing, plates were transferred to a plate reader and fluorescence data collected by monitoring emission at 598 nm throughout a 20 min reaction (Figure 4A). These data revealed an enzyme-dependent increase in fluorescence that reached a plateau after 20 min, with background subtracted from the 0 nM control. The interpretation that this increase in fluorescence intensity was a measure of DNA incision and subsequent strand separation was confirmed by analyzing the DNA reactants and products using changes in the electrophoretic mobility of these DNAs (Figure 4B). As analyzed by polyacrylamide gel electrophoresis, the cleavage reaction proceeded more quickly than that revealed by the increase in fluorescent signal. This result is likely due to a slow dissociation of the enzyme from the cleaved substrate, or a slow dissociation of the released fragment.

### Quantitative high-throughput screening (HTS) of small molecule libraries

**Figure 5:**
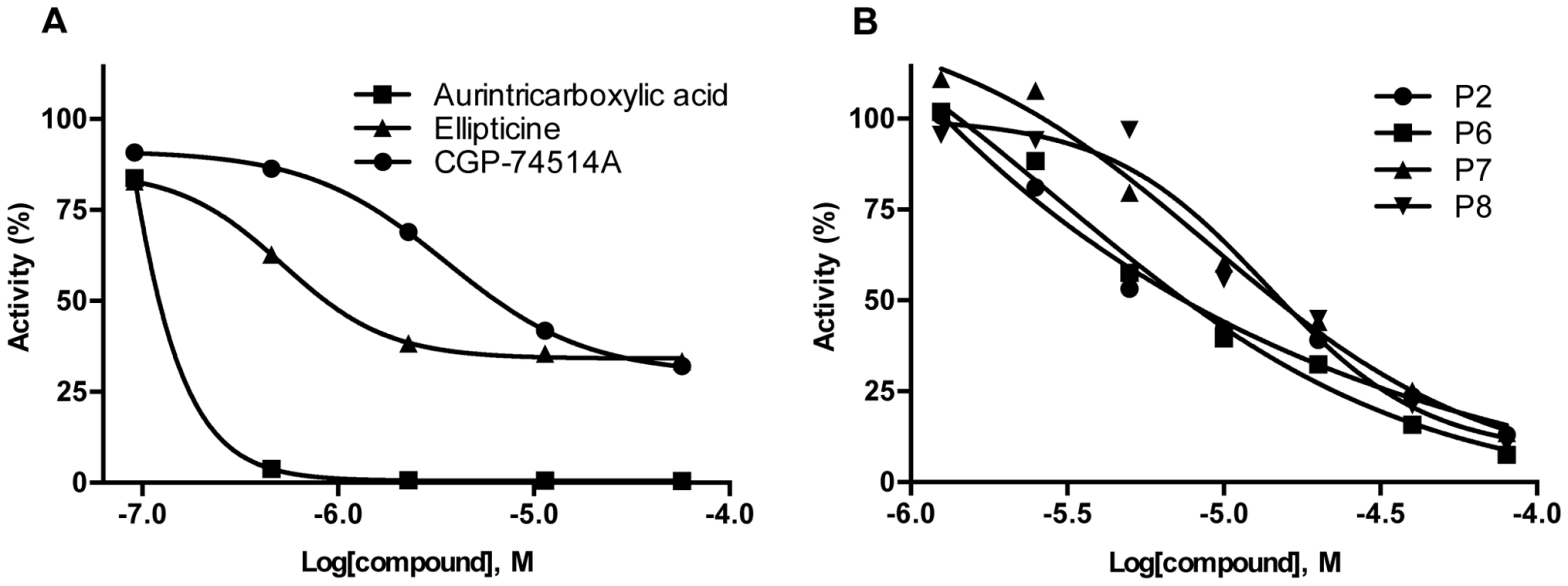
High-throughput screen of selected small molecule libraries. A) Inhibition of NEIL1 activity in the HTS of the LOPAC library by aurintricarboxylic acid (squares), ellipticine (triangles), and CGP-74514A (circles). B) Inhibition of NEIL1 activity by purine analogs P2 (circles), P6 (squares), P7 (triangles), and P8 (inverted triangles).

Having established the general principle for a glycosylase screening assay, further assay development was undertaken to enable HTS in which the reaction volumes were reduced by 5-fold to a total of 4 µL. Initially, the library of pharmacologically active compounds (LOPAC^1280^) was screened for inhibitors of NEIL1 as a determination of the feasibility of this high-throughput method. The calculated average Z′ score of 0.82 demonstrated the robustness of the screen. All compounds were screened at 5 different concentrations that allowed for the initial estimation of inhibitory potency (IC_50_) values, as well as an evaluation of the shape of the response curves. In addition to the anticipated inhibitors (known DNA intercalators, such as ellipticine (IC_50_ = 2 µM)) and other small molecules that have been previously established to be promiscuous inhibitors (such as aurintricarboxylic acid (IC_50_<1 µM)), one interesting hit from this screen was CGP-74514A (NCGC ID is NCGC00015229) (Figure 5A). CGP-74514A is a known small molecule inhibitor of Cdk1 in which the structure of this molecule is based on a purine ring backbone.

**Figure 6:**
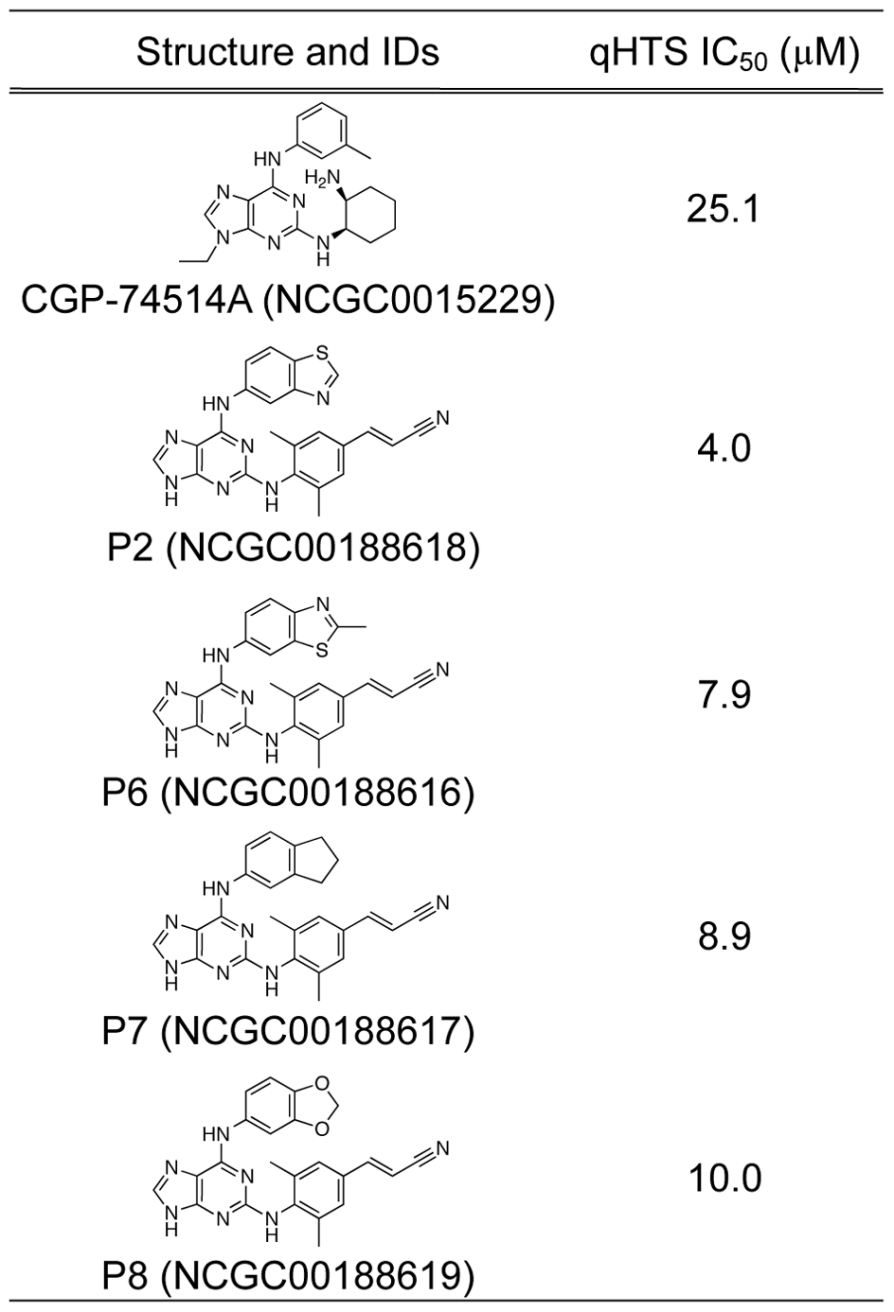
Purine analogs that inhibit NEIL1.

**Figure 7:**
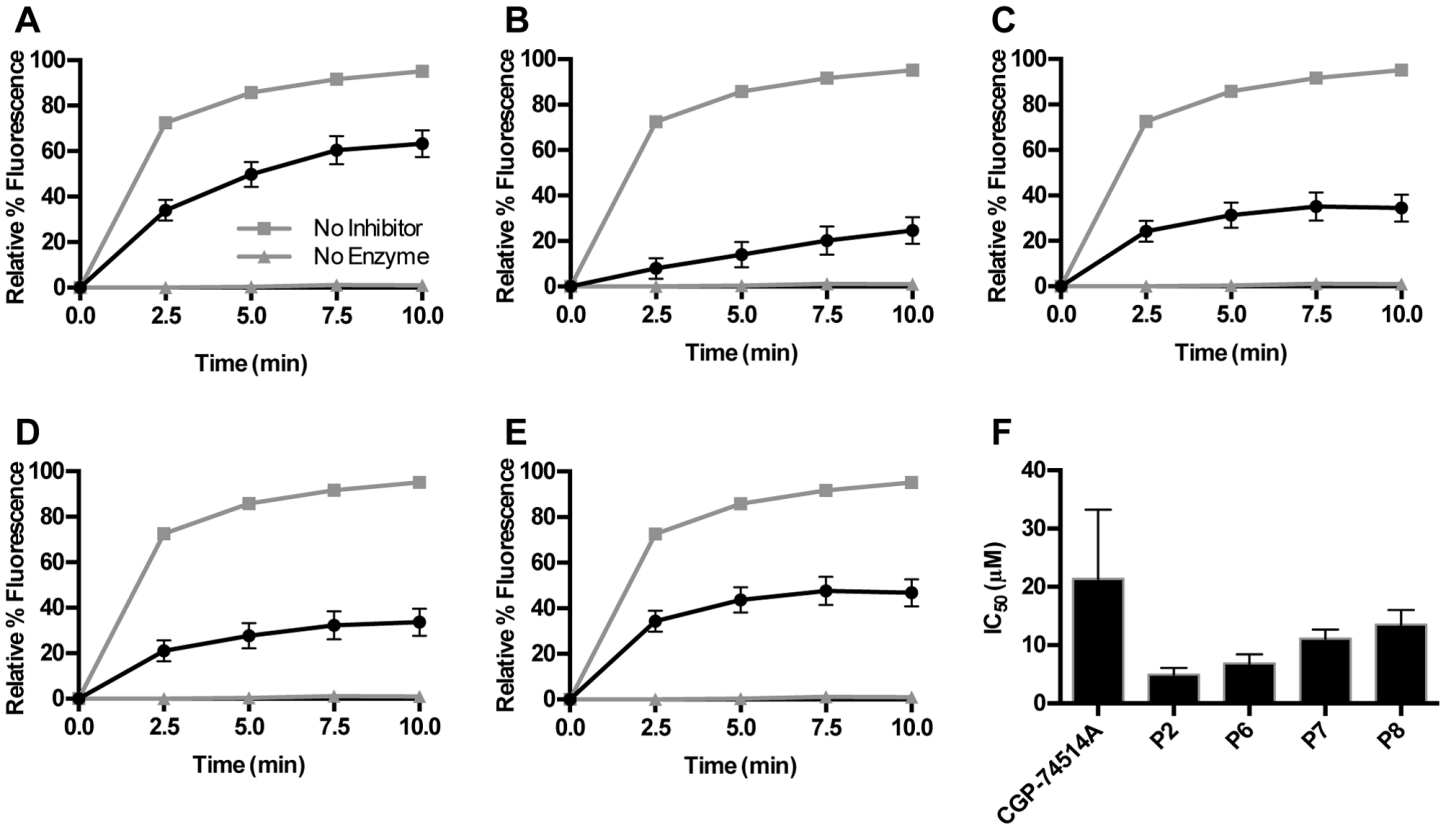
Inhibition of NEIL1 activity with 10 µM concentrations of select compounds. A) CGP-74514A, B) P2, C) P6, D) P7, E) P8 compared to no inhibitor (gray squares) or no enzyme (gray triangles). F) IC_50_ values determined during validation of HTS.

Since the prefered targets of NEIL1 are modified purines, inhibition by CGP-74514A suggested that other purines may also have inhibitory properties. An additional library consisting of ≈1400 modified purine analogs was cherry-picked from other NCGC collections and screened over a 5 concentration range. The results of this screen yielded 42 potential inhibitors with IC_50_ values in the range of 5–50 µM (Figure S1). For triaging purposes, these compounds were rescreened using the 384-well format and a subset of these were subsequently confirmed by incision of oligodeoxynucleotides containing site-specific base lesions as analyzed by mobility differences through polyacrylamide gels. In addition to CGP-74514A, a total of four more compounds (P2 NCGC000188618, P6 NCGC000188616, P7 NCGC000188617 and P8 NCGC000188619) displayed favorable IC_50_s (Figure 5B, Figure 6 and Figure S1). These were further characterized by fluorescent-based kinetic inhibition analyses. Two additional purine analogs that were poor inhibitors in the HTS (P11 NCGC ID = NCGC000182914 and P19 NCGC ID = MLS001126460) were also re-analyzed and shown to have IC_50_s above 50 µM (data not shown). Single concentration (10 µM purine analog) kinetic analyses showed significant inhibition and 7 concentration inhibition curves revealed IC_50_s between 5–14 µM (Figure 7).

### Inhibition of NEIL1 activity on γ-irradiated DNA as measured by GC-MS/MS

**Figure 8:**
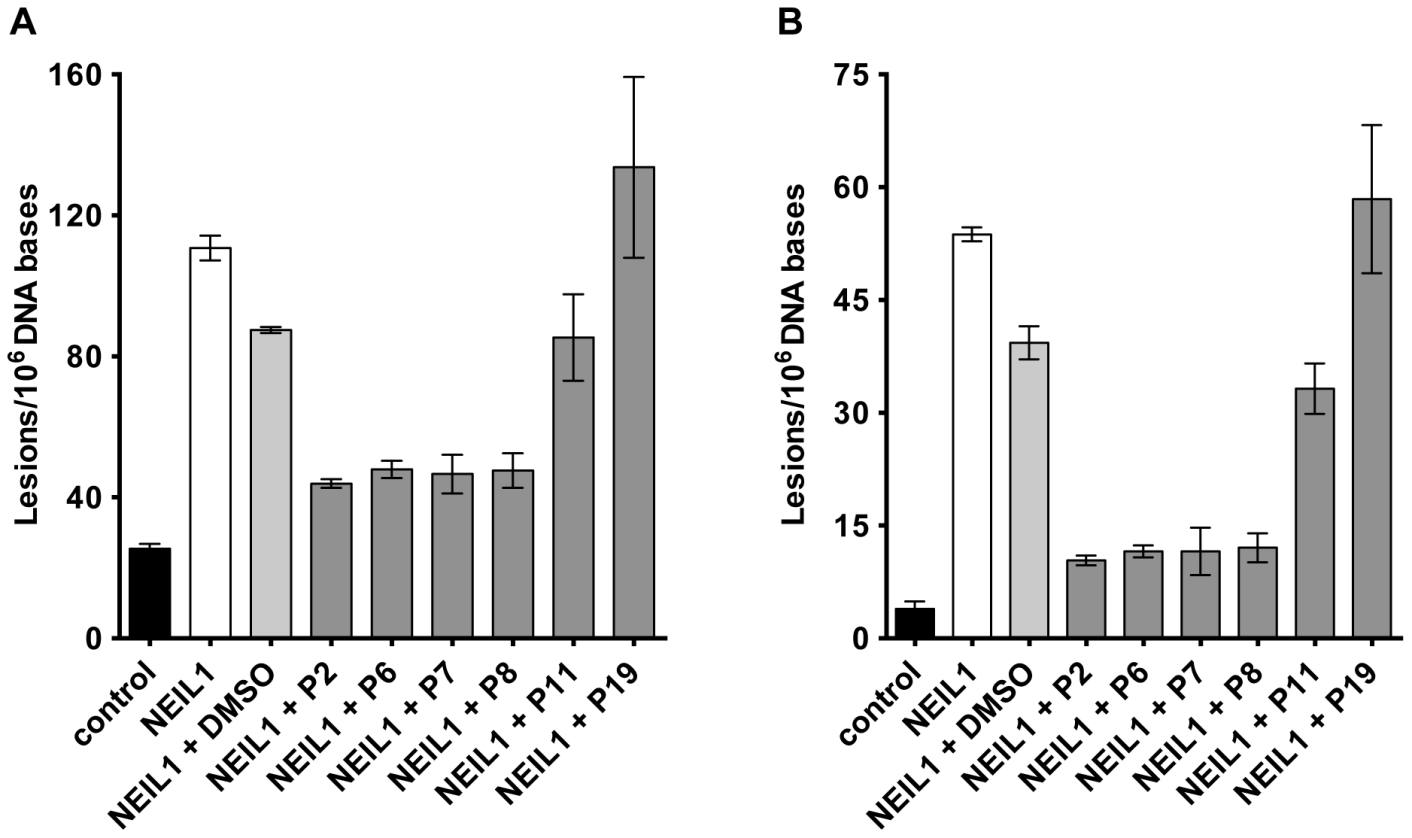
Determination of NEIL1 inhibition by GS-MS/MS. Levels of A) FapyGua and B) FapyAde released from γ-irradiated DNA by NEIL1 in the absence or presence of various inhibitors.

Although Gh and Sp are excellent substrates for NEIL1, these are likely not to be the biologically relevant substrates. Thus, to determine if the compounds that were identified above were effective in the inhibition of NEIL1 against its biologically relevant substrates FapyAde and FapyGua [5], [6], γ-irradiated calf thymus DNA was used as a substrate. The excision by NEIL1 of these substrates from DNA was measured by GC-MS/MS with isotope-dilution. Figure 8 illustrates the excision of FapyAde and FapyGua by NEIL1 from γ-irradiated calf thymus DNA in the absence or presence of inhibitors. NEIL1 exhibited strong activity for excision of both lesions in agreement with previously published data [2], [3], [4], [5], [6]. Inhibitors P2, P6, P7 and P8 significantly reduced the activity of NEIL1 (*p*<0.005). Some inhibition by DMSO alone was also observed when compared with excision by NEIL1 alone (*p* = 0.01). However, when compared to DMSO, the inhibitions by P2, P6, P7 and P8 were highly significant (*p*<0.005). To a lesser extent, P11 (NCGC ID = NCGC000182914) inhibited FapyAde excision (*p* = 0.0046); but not FapyGua excision (*p* = 0.08). These data suggest that the observed inhibition of FapyAde excision by P11 may have been the effect of DMSO, not the inhibitor itself. Inhibitor P19 exhibited no inhibition. Statistical analyses of these data were performed using the GraphPad Prism 5.04 software (La Jolla, CA, USA) and two-tailed nonparametric Mann Whitney test with Gaussian approximation and confidence interval of 95%. A p-value<0.05 was assumed to correspond to statistical significance.

### Purine analog inhibitors of NEIL1 inhibit other glycosylases involved in the recognition of other oxidatively-induced DNA damage

**Figure 9:**
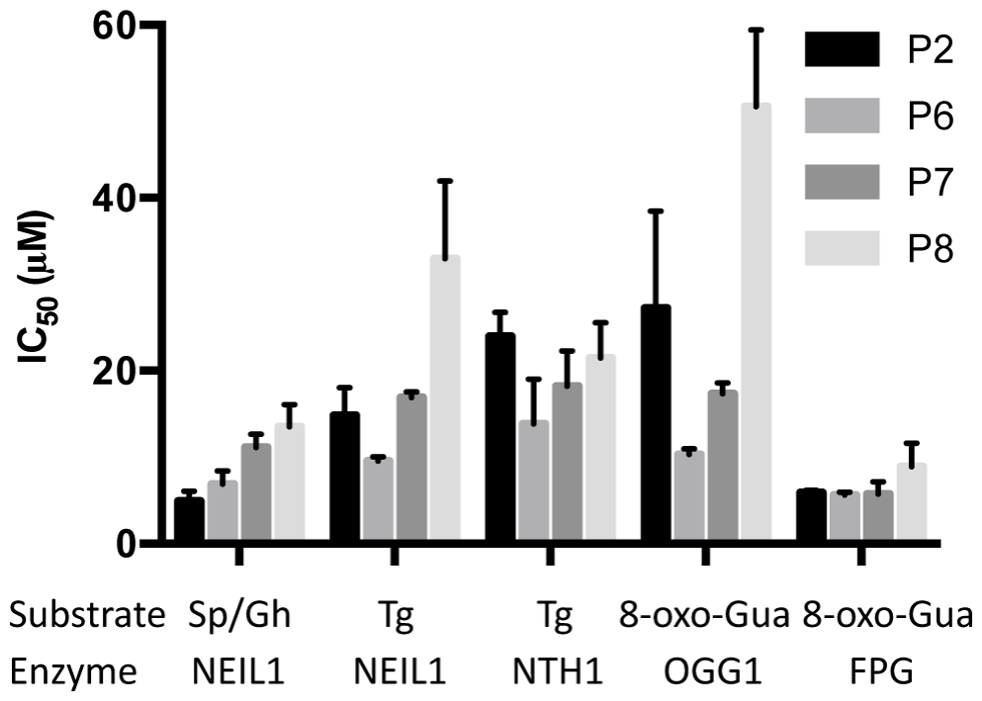
Specificity of various purine inhibitors for different glycosylase-substrate combinations as determined by IC_50_ value.

In humans, several glycosylases, including OGG1, NTH1, NEIL2, and NEIL3 may participate in the removal of a wide variety of oxidized bases. Thus, although several of the purine analogs inhibited the ability of NEIL1 to remove Sp, Gh, FapyAde and FapyGua lesions, these assays were not informative concerning their specificity for NEIL1 and did not address whether these molecules could inhibit other DNA glycosylase/AP lyases that participate in the removal of oxidatively-induced DNA lesions. To address this question, assays were designed to measure inhibition of OGG1 and NTH1, as well as the *Escherichia coli* glycosylase-AP lyase, FPG. In order to compare the relative efficiencies of inhibition between NEIL1 and these other glycosylase/AP lyases, it was necessary to identify common DNA substrates and pair-wise analyze inhibition data. To compare inhibition of NEIL1 versus NTH1, oligodeoxynucleotides were synthesized that contained a site-specific thymine glycol (TG) adduct, while comparative inhibition data for OGG1 and FPG were collected using an oligodeoxynucleotide containing a site-specific 8-oxo-Gua. In control reactions (no inhibitor added), each enzyme generated the expected incised products, confirming the validity of the assay. Since compounds P2, P6, P7, and P8 showed significant inhibition of NEIL1 activity on Sp/Gh during the HTS, they were used to determine the IC_50_ values of purine inhibitors toward each of the base excision repair glycosylases (Figure 9). IC_50_ values ranged from 5–50 µM, with FPG and NEIL1 being the most severely inhibited for all the compounds tested. Specifically, using the same 8-oxo-Gua substrate, this series of compounds elicited a 2–5 fold more potent IC_50_ when used to inhibit FPG relative to OGG1. In general, P8 showed the poorest inhibition for all enzymes except for NTH1. Furthermore, although not always the most potent inhibitor, P6 appears to be the most general inhibitor, with the IC_50_ values for all four enzymes ranging between 9 µM and 28 µM. Additionally, experiments were designed to test whether compounds P2, P6, P7, and P8 could inhibit a pyrimidine dimer-specific glycosylase/AP lyase, T4 pyrimidine dimer DNA glycosylase (T4-pdg) that also has an associated AP lyase activity, using an UV-irradiated plasmid nicking assay. T4-pdg was preincubated with the highest concentration of each inhibitor (40 µM) and subsequently incubated for 15 min with plasmid DNAs containing ∼10 dimers per molecule. Following triplicate independent analyses by agarose gel electrophoresis, analyses of the extent of nicking revealed that P2, P6, P7, and P8 inhibited activity by <1%, 32%, 13%, and 14%, respectively. Since it was not possible to increase these concentrations significantly higher to determine an IC_50_ for any of these compounds, it was concluded that these molecules are very poor inhibitors of the T4-pdg. Overall, these data suggest that due to the relatively low affinity of these compounds for these enzymes and the modest differences in rank order and substrate specificities, future derivatization of purine analogs may provide specificity for individual DNA glycosylases.

### Specificity of the inhibition of NEIL1

Given that several of the purine analogs inhibited selected DNA glycosylases, there are several possible mechanisms by which these small molecules could inhibit strand scission. Theoretically, it could bind in the active site or at an allosteric site and prevent the cleavage of the lesion's glycosidic bond, or alternatively, it may allow glycosidic bond cleavage, producing an abasic site, but inhibit the lyase activity. Alternatively, it could interact with the DNA in such a way that the enzyme either does not bind or is unable to locate and cleave at the site of the lesion.

**Figure 10:**
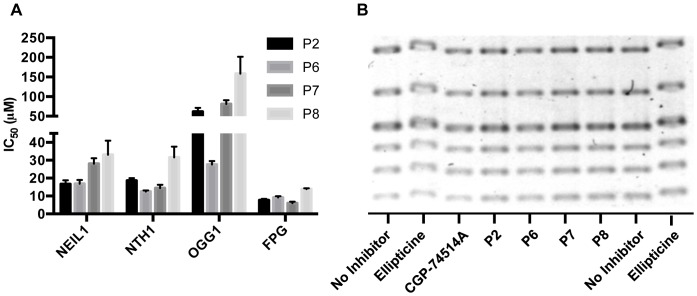
Specificity of the inhibition of NEIL1. A) Inhibition of the lyase activity of various bifunctional glycosylases with purine analogs. B) DNA ladder gel shift assay with addition of various inhibitors or ellipticine control.

To test for inhibition of lyase activity, the DNA substrate was changed to an oligodeoxynucleotide containing an AP site. In the absence of inhibitor molecules, this substrate reacted rapidly with NEIL1, NTH1, OGG1 or FPG to generate either a β or a β/δ elimination product. Although the IC_50_ values for these reactions were higher than that measured for the glycosylase reaction, in general, the addition of the purine inhibitors resulted in a significant reduction in the cleavage reaction (Figure 10A). The major exception to this was observed for OGG1, in which it showed very little lyase inhibition with all compounds except P6. This could be potentially explained by P2, P7, and P8 not interfering with OGG1's catalytic residues Lys249 or Asp268, but rather the Lys249-Cys253 dipole that recognizes the lesion [39], producing a lyase-only activity similar to a C253A mutant [40]. Overall, these data suggest that these purine analogs preferentially act to block the glycosylase, and to a lesser extent, the lyase step. This fits with the hypothesis that purine analogs would fit inside the active site of these glycosylases, sterically hindering the residues required for activity.

To assay whether these inhibitors could block NEIL1 activity through DNA intercalation, a DNA ladder gel-shift assay was performed, with ellipticine serving as a positive control [33]. A duplex DNA ladder was incubated with each inhibitor, and analyzed for electrophoretic mobility shift. As shown in Figure 10B, while ellipticine showed a major reduction in the electrophoretic mobility of the DNA ladder, none of the purine analog inhibitors produced such a shift. These data indicate that the inhibitors had minimal, if any interaction with the DNA, and instead produced their effect through interaction with the enzymes. These data are also consistent with the mass spectrometry data in which there are vast excesses of undamaged DNAs present in those reactions.

### Cytotoxicity of purine analogs

Although the overall goal of this investigation was to design and reduce-to-practice a reliable, high-throughput fluorescence-based screening assay for inhibitors of DNA glycosylases, it was also of interest to determine if these compounds exhibited cellular toxicities that would be indicative of cellular uptake and interference with critical cellular functions. To experimentally address this question, mouse embryo fibroblasts from wild-type and *Neil*1 knockout mice were treated with increasing concentrations of P2, P6, P7, and P8. Cell survival was measured after a 72 h exposure. For all four compounds, the 37% survival was greater in the *Neil*1 knockout cells relative to the wild-type. The D_37_ ratios for WT: *Neil*1 were as follows: P2: 6∶10 µM; P6: 5∶8 µM; P7: 12∶15 µM; and P8: 7∶11 µM, respectively. The trend of these data are generally consistent with the relative IC_50_ values for each of the compounds and the values are within 2-fold of the calculated IC_50_s for all four compounds tested. Although these data reveal that in the absence of cellular NEIL1, the relative cytotoxicities of these compounds is reduced, the mechanism(s) by which these purine analogs kill cells is unknown. In addition to modulating BER, there are potentially many other candidate enzymes that may be compromised by the introduction of these compounds, including but not limited to kinases, DNA and RNA polymerases, other DNA repair complexes or helicases. However, given that the concentrations of these compounds are several orders of magnitude higher than what would be considered as a pharmacologically-relevant dose, detailed mechanistic analyses of the current set of compounds is highly premature. Such studies and the development of appropriate cell culture and animal models will need to be generated concomitantly with the evolution of higher affinity, greater specificity molecules.

### Conclusions

Overall, the data presented herein form the foundation for future drug discovery for the entire family of DNA glycosylases. Even though the current assay design relied on the strand incision activity following the cleavage of the glycosyl bond through β or β/δ elimination reactions, for DNA glycosylases that only cleave the glycosyl bond leaving an AP site, the assay could be readily modified to include a secondary biochemical or chemical reaction to cleave the phosphodiester backbone at that site. Thus, the small molecule inhibitors that were identified in this limited screen serve as a proof-of-concept for the initial phase of the drug discovery process. However, the molecules identified herein do not necessarily represent the only lead compounds for future medicinal chemistry development. This cautionary note is exemplified by the observation that while NEIL1 is primarily associated with recognition of damaged purines, NTH1 covers a broad spectrum of saturated pyrimidines, and both NEIL1 and NTH1 were similarly inhibited by many of these small molecules. Future experimental strategies will include the development of cell biology-based readouts for DNA glycosylase inhibitors and the screening of additional libraries for higher affinity inhibitors with greater specificity.

## Supporting Information

Figure S1 **Compounds of interest from the HTS of a purine analog library.**
(PDF)
