# Supplementary material for: Inhibition of DNA Glycosylases via Small Molecule Purine Analogs
Source: PLoS One. 2013 Dec 9;8(12):e81667. doi: 10.1371/journal.pone.0081667 (PMC3857224; doi:10.1371/journal.pone.0081667)
Supplement: Figure S1 — Compounds of interest from the HTS of a purine analog library. (PDF) [file pone.0081667.s001.pdf]

Supplementary 1

Purine ID	NCGC ID	Structure	qHTS IC_{50} (μM)	Gel Assay IC_{50} (μM)
P1	NCGC00188025	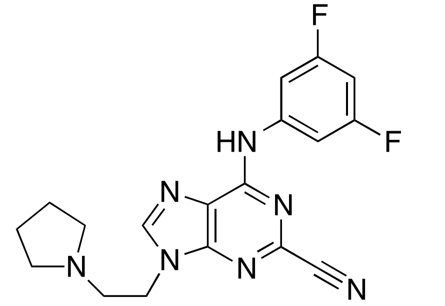	3.6	> 50
P2	NCGC00188618	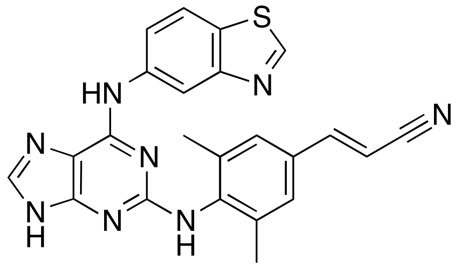	4.0	28
P3	MLS000565379	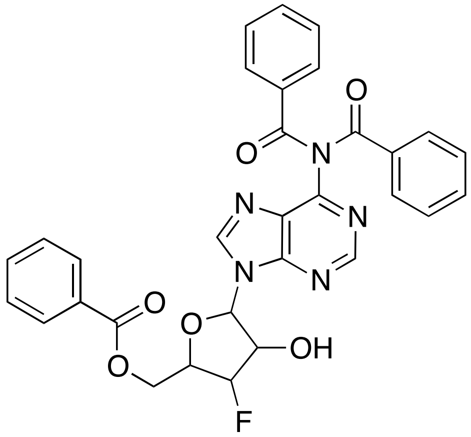	3.2	> 50
P4	MLS000696940	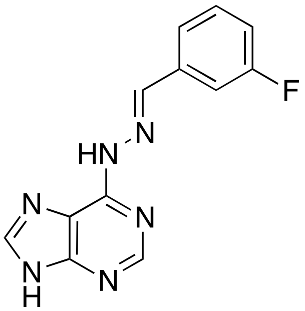	8.9	> 50

Purine ID	NCGC ID	Structure	qHTS IC_{50} (μM)	Gel Assay IC_{50} (μM)
P5	NCGC00188028	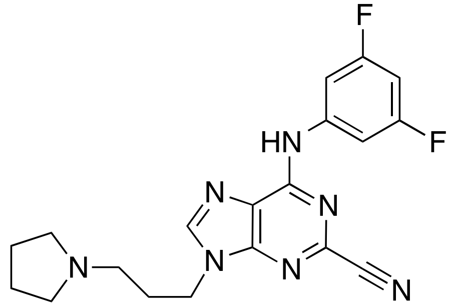	7.9	> 50
P6	NCGC00188616	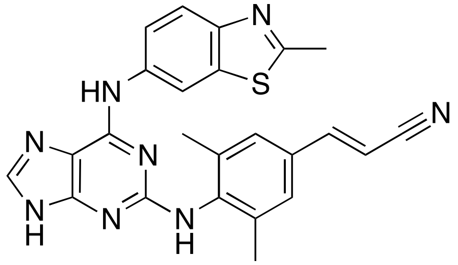	7.9	9.2
P7	NCGC00188617	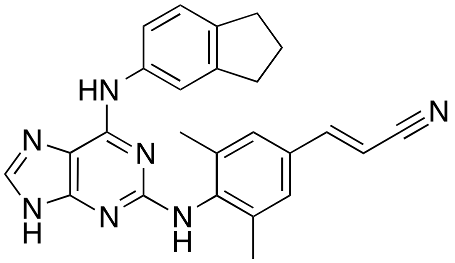	8.9	11.7
P8	NCGC00188619	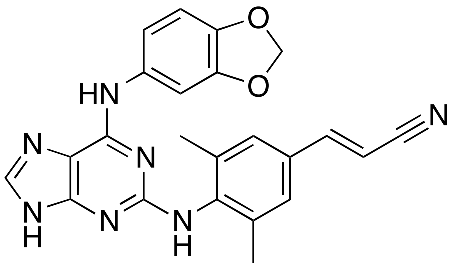	10.0	15.2
P10	NCGC00182112	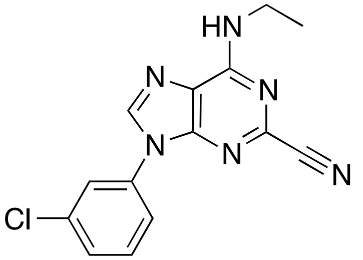	10.0	> 50

Purine ID	NCGC ID	Structure	qHTS IC_{50} (μM)	Gel Assay IC_{50} (μM)
P11	NCGC00182914	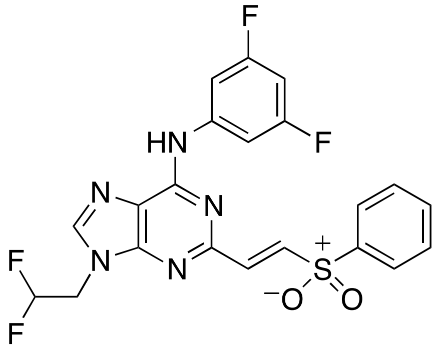	11.2	> 20
P12	MLS002232283	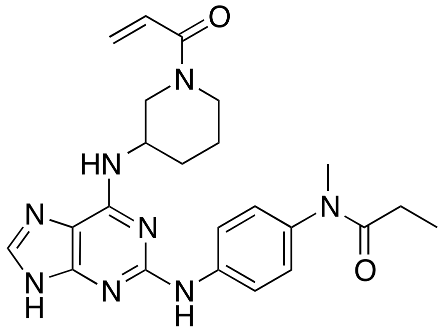	11.2	> 20
P13	MLS000911541	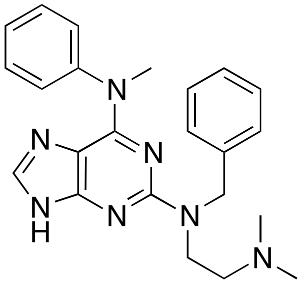	12.6	> 50
P14	MLS002632768	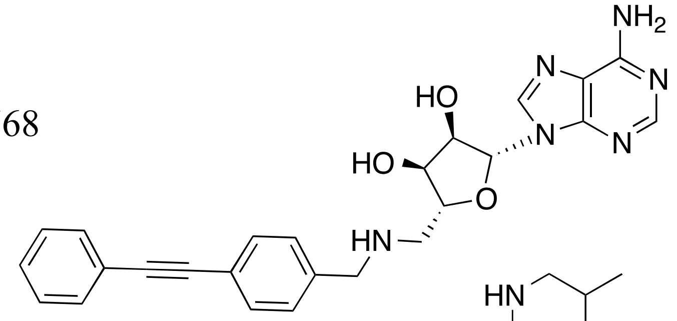	12.6	> 50
P15	MLS003120811	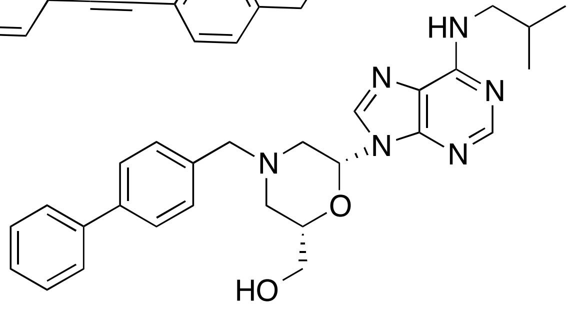	14.1	> 50

Purine ID	NCGC ID	Structure	qHTS IC_{50} (μM)	Gel Assay IC_{50} (μM)
P16	MLS003120816	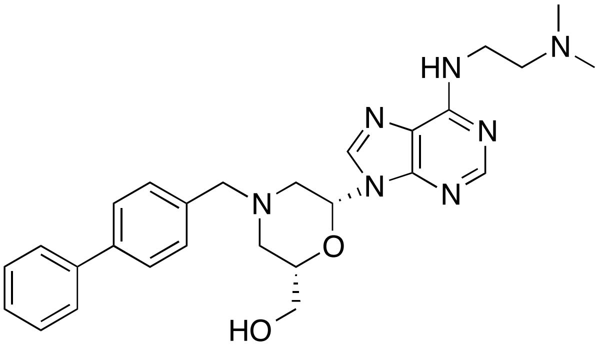	15.8	> 50
P17	MLS001029196	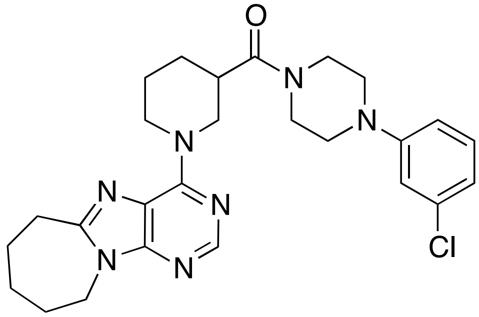	15.8	> 50
P18	NCGC00188620	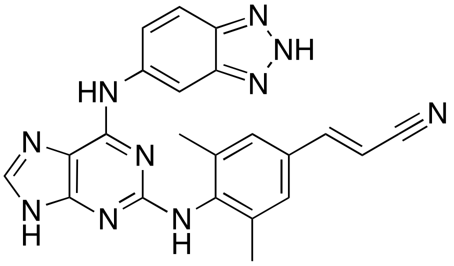	17.8	> 50
P19	MLS001126460	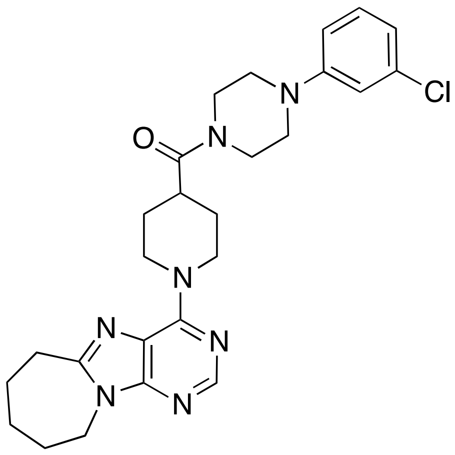	20.0	> 20

Purine ID	NCGC ID	Structure	qHTS IC$_{50}$ (μM)	Gel Assay IC$_{50}$ (μM)
P20	MLS003105994	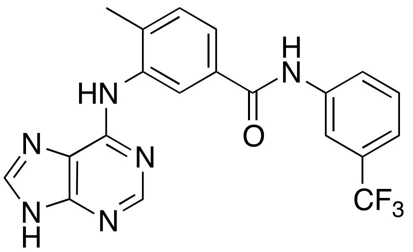	22.4	> 50
P21	MLS002477235	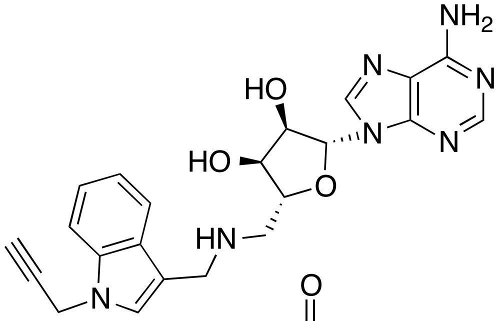	25.1	> 50
P22	MLS001029126	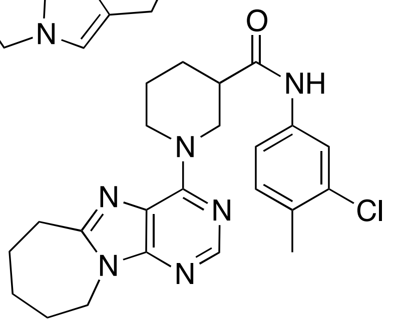	25.1	> 50
P23	NCGC00182888	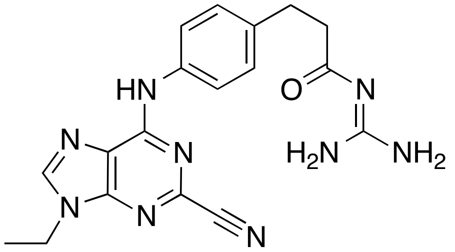	28.2	> 50
P24	MLS000419189	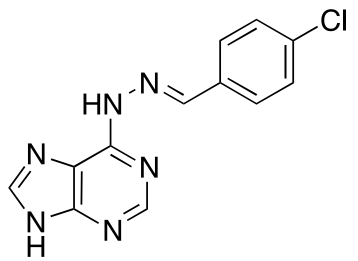	28.2	> 50

Purine ID	NCGC ID	Structure	qHTS IC_{50} (μM)	Gel Assay IC_{50} (μM)
P25	MLS002632722	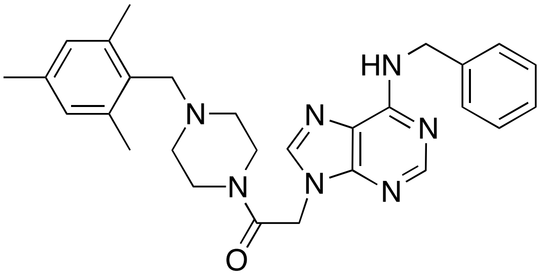	31.6	> 20
P26	MLS000101201	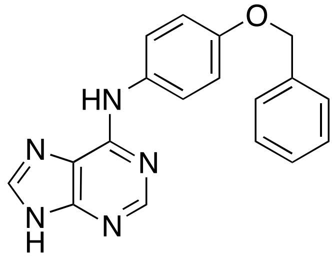	31.6	> 20
P27	MLS001029138	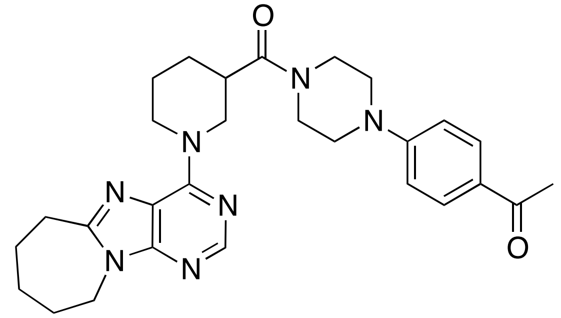	0.6	> 50
P28	MLS001124185	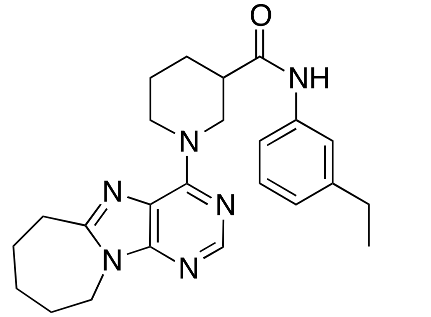	0.7	> 20
P29	MLS003120675	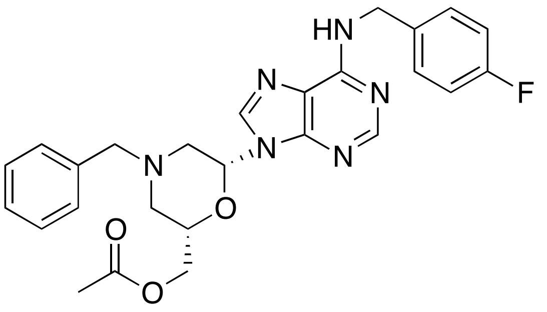	0.9	> 50

Purine ID	NCGC ID	Structure	qHTS IC_{50} (μM)	Gel Assay IC_{50} (μM)
P30	NCGC00024978	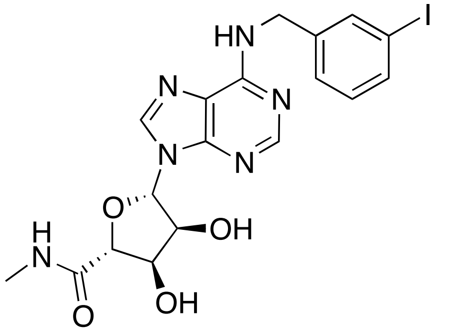	1.8	> 50
P31	MLS001029155	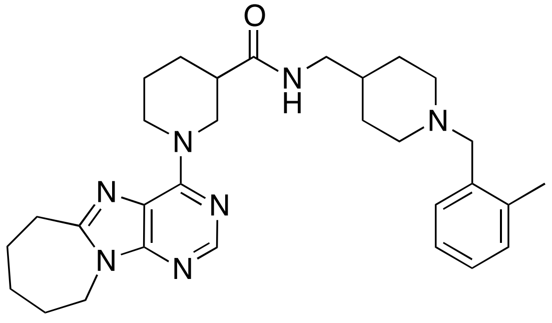	7.1	> 50
P32	MLS000911550	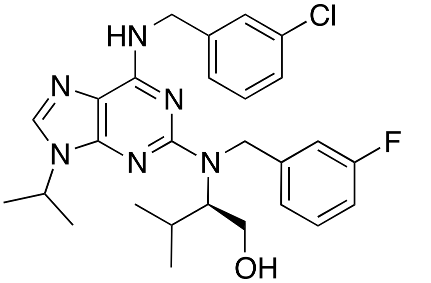	7.1	19.3
P33	MLS000911556	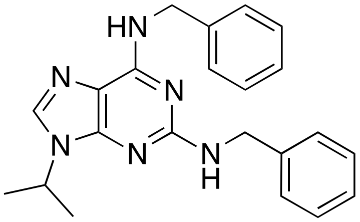	7.9	> 50
P34	MLS003271226	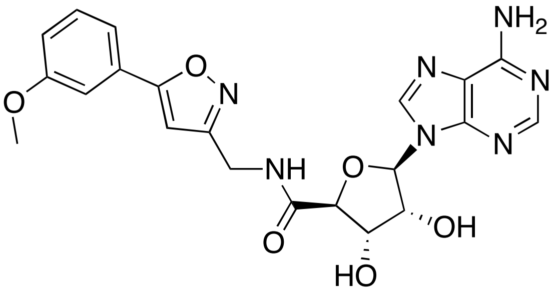	10.0	> 50

Purine ID	NCGC ID	Structure	qHTS IC_{50} (μM)	Gel Assay IC_{50} (μM)
P35	MLS003391039	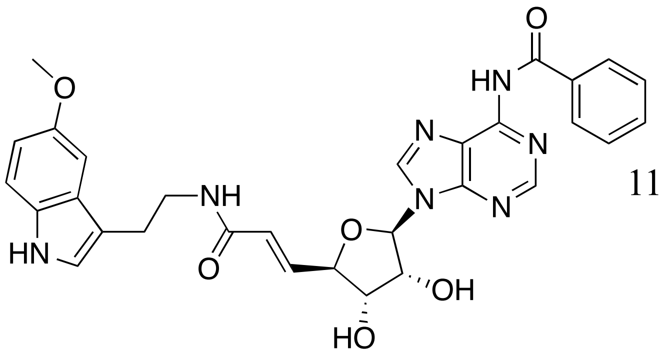	11.2	> 50
P36	MLS001183408	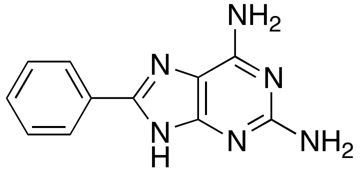	15.5	> 50
P37	MLS001029140	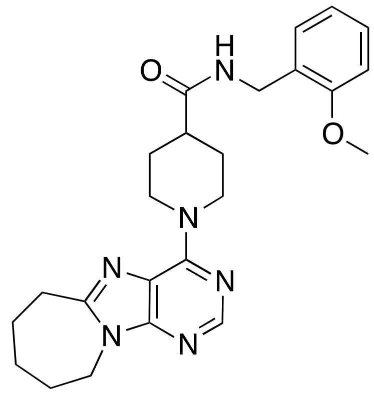	10.0	> 50
P38	MLS003120763	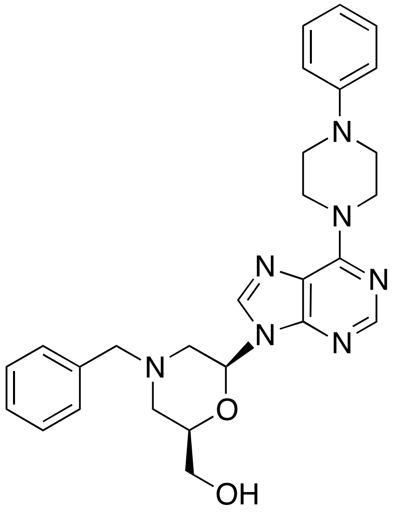	0.3	> 50

Purine ID	NCGC ID	Structure	qHTS IC_{50} (μM)	Gel Assay IC_{50} (μM)
P39	MLS000580432	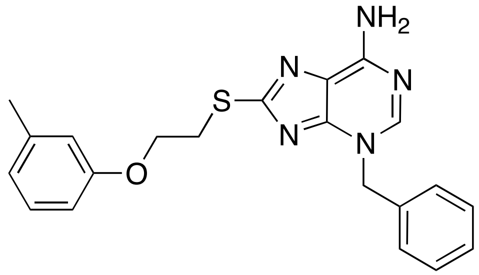	0.6	> 50
P40	MLS000911549	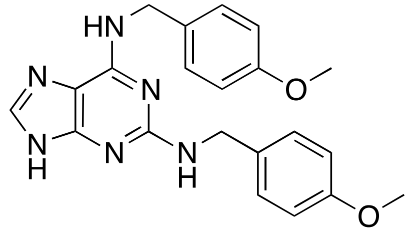	0.6	> 50
P41	MLS001029127	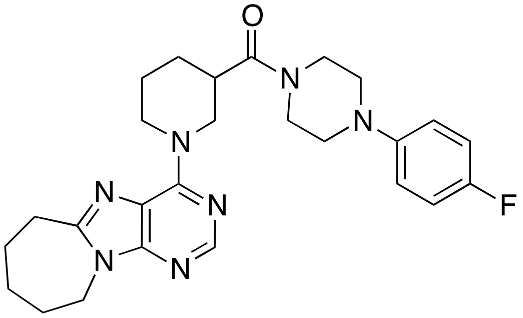	0.8	> 50
P42	MLS001199880	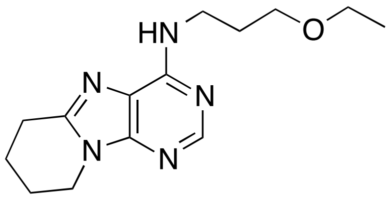	0.8	> 50
